# Evolution of the human brain: when bigger is better

**DOI:** 10.3389/fnana.2014.00015

**Published:** 2014-03-27

**Authors:** Michel A. Hofman

**Affiliations:** Netherlands Institute for Neuroscience, Royal Netherlands Academy of Arts and SciencesAmsterdam, Netherlands

**Keywords:** brain evolution, cerebral cortex, cortical design, neural networks, information processing, intelligence, primates, human neocortex

## Abstract

Comparative studies of the brain in mammals suggest that there are general architectural principles governing its growth and evolutionary development. We are beginning to understand the geometric, biophysical and energy constraints that have governed the evolution and functional organization of the brain and its underlying neuronal network. The object of this review is to present current perspectives on primate brain evolution, especially in humans, and to examine some hypothetical organizing principles that underlie the brain's complex organization. Some of the design principles and operational modes that underlie the information processing capacity of the cerebral cortex in primates will be explored. It is shown that the development of the cortex coordinates folding with connectivity in a way that produces smaller and faster brains, then otherwise would have been possible. In view of the central importance placed on brain evolution in explaining the success of our own species, one may wonder whether there are physical limits that constrain its processing power and evolutionary potential. It will be argued that at a brain size of about 3500 cm^3^, corresponding to a brain volume two to three times that of modern man, the brain seems to reach its maximum processing capacity. The larger the brain grows beyond this critical size, the less efficient it will become, thus limiting any improvement in cognitive power.

## Introduction

The human brain contains about 100 billion neurons, more than 100,000 km of interconnections, and has an estimated storage capacity of 1.25 × 10^12^ bytes (Cherniak, 1990; Hofman, 2012). These impressive numbers have led to the idea that our cognitive capabilities are virtually without limit. The human brain, however, has evolved from a set of underlying structures that constrain its size, and the amount of information it can store and process. If the ability of an organism to process information about its environment is a driving force behind evolution, then the more information a system, such as the brain, receives, and the faster it can process this information, the more adequately it will be able to respond to environmental challenges and the better will be its chances of survival (Macphail and Bolhuis, 2001; Roth and Dicke, 2012; Hofman, 2014). The limit to any intelligent system therefore lies in its abilities to process and integrate large amounts of sensory information and to compare these signals with as many memory states as possible, and all that in a minimum of time. It implies that the functional capacity of a neuronal structure is inherently limited by its neural architecture and signal processing time (see e.g., Laughlin and Sejnowski, 2003; Buzsáki et al., 2013). The object of this review is to present current perspectives on primate brain evolution, especially in humans, and to examine some hypothetical organizing principles that underlie the brain's complex organization. Some of the design principles and operational modes that underlie the information processing capacity of the cerebral cortex in primates will be explored, and it will be argued that with the evolution of the human brain we have nearly reached the limits of biological intelligence.

## Principles of brain evolution

If we assume that biological intelligence in higher organisms is the product of processes of complex sensory information processing and mental faculties, responsible for the planning, execution and evaluation of intelligent behavior, variations among species in intelligence must in principle be observable in the neural substrate. In higher organisms, especially in primates, the complexity of the neural circuitry of the cerebral cortex is considered to be the neural correlate of the brain's coherence and predictive power, and, thus, a measure of intelligence.

**Figure 1:**
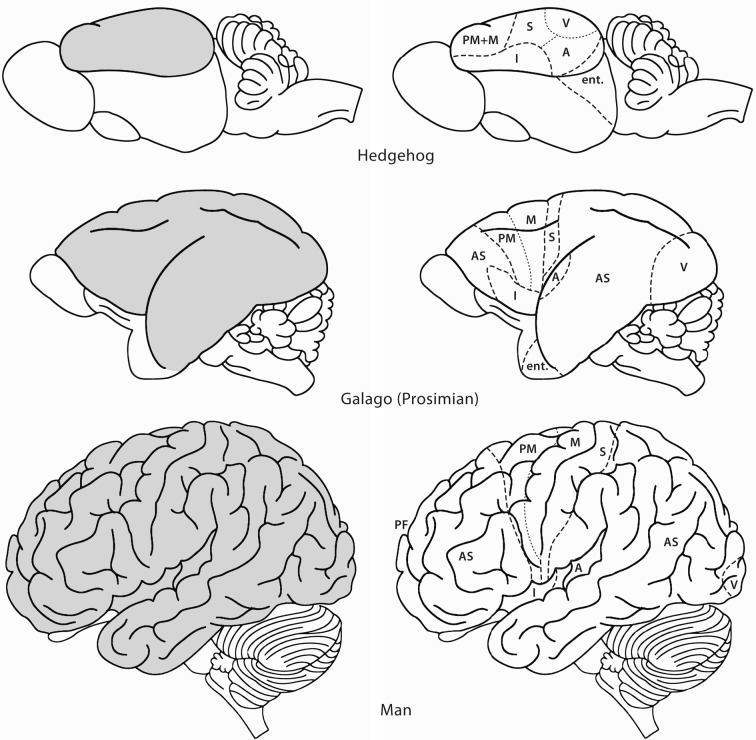
**Lateral views of the brains of some mammals to show the evolutionary development of the neocortex (gray).** In the hedgehog almost the entire neocortex is occupied by sensory and motor areas. In the prosimian *Galago* the sensory cortical areas are separated by an area occupied by association cortex (AS). A second area of association cortex is found in front of the motor cortex. In man these anterior and posterior association areas are strongly developed. A, primary auditory cortex; AS, association cortex; Ent, entorhinal cortex; I, insula; M, primary motor cortex; PF, prefrontal cortex; PM, premotor cortex; S, primary somatosensory cortex; V, primary visual cortex. Modified with permission from Nieuwenhuys (1994).

The evolutionary expansion of the cerebral cortex, indeed, is among the most distinctive morphological features of mammalian brains. Particularly in species with large brains, and most notably in great apes and marine mammals, the brain becomes disproportionately composed of this cortical structure (Northcutt and Kaas, 1995; Striedter, 2005; Aboitiz and Montiel, 2012; Sherwood et al., 2012; Figure 1). The volume of cortical gray matter, for example, expressed as a percentage of total brain volume increases from about 25% for insectivores to 50% for humans (Frahm et al., 1982; Hofman, 1988), whereas the relative size of the entire cerebral cortex (including white matter) goes from 40% in mice to about 80% in humans (Hofman, 1988; Azevedo et al., 2009; Herculano-Houzel, 2009, 2012).

On the other hand, the relative size of the cerebellum remains constant across phylogenetic groups, occupying about 10–15% of the total brain mass in different orders (Stephan et al., 1981). Comparative studies among four mammalian orders, including primates, have recently revealed that the absolute neuronal composition in the cerebral cortex covaries significantly with that of the cerebellum (Herculano-Houzel et al., 2008; Lent et al., 2012), showing that these two brain structures display coordinated growth during phylogenesis in mammals.

Such a coordinated evolution of the cerebral cortex and cerebellum fits well with the recent clinical and experimental evidence suggesting an important role of the cerebellum in cognitive and affective functions, in close connection with cortical associative areas (for reviews, see Schmahmann, 2010; MacLeod, 2012). Although the cerebral cortex is not the only brain structure which was selected for in evolution for greater growth, as a result of growing environmental pressure for more sophisticated cognitive abilities, it has played a key role in the evolution of intelligence.

### Evolution of the cerebral cortex

It is now well established that the cerebral cortex forms as a smooth sheet populated by neurons that proliferate at the ventricular surface and migrate outwards along radial glial fibers (for reviews, see Cheung et al., 2007; Rakic, 2009). Differences in the duration of neurogenesis, which increases more rapidly with brain size for the cerebral cortex than for subcortical areas (Finlay et al., 2001; Charvet and Finlay, 2012), lead to a systematic increase in the ratio of the cortical to subcortical regions. Whereas in small brained species the cortical volume expands by virtue of a combined increase in surface area and cortical thickness, the increase of the cortical volume in species with a brain size of more than 3–4 cm^3^ is almost entirely due to a disproportionate expansion of the cortical surface area (Hofman, 1989). It is the increase of the cortical surface area beyond that expected for geometrically similar objects of different volumes which creates the need to cortical folding (Jerison, 1982; Todd, 1986; Hofman, 1989, 2012, Figure 2).

**Figure 2:**
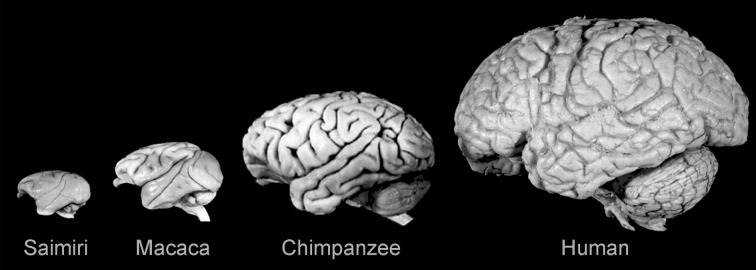
**Lateral views of the brains of some anthropoid primates showing the evolutionary expansion of the neocortex.** Note the diverse configurations and gyral and sulcal patterns. *Saimiri sciureus*: *E* = 22 g; *Macaca mulatta*: *E* = 95 g; *Pan troglodytes*: *E* = 420 g; *Homo sapiens*: *E* = 1350 g. Reproduced with permission from Hofman (2007).

Consequently, the brains of larger species, like primates, are not well described by the ideal constructs of Euclidean geometry. Mandelbrot (1982) coined the word “fractal” to identify this group of complex geometric forms and developed the concept of fractal scaling to describe their organized variability. An important feature of fractal objects is that they are invariant, in a statistical sense, over a wide range of scales, a property that is known as scaling (for a review, see Hofman, 2012).

In mammals with convoluted brains, among which are almost all primates, the cortical surface area, rather than being proportional to the 2/3 power of geometric similarity, is nearly a linear function of brain volume (Hofman, 1985a, 1989). It means that if a mouse brain (volume = 0.5 cm^3^) were scaled up as the two-thirds power to the size of the human brain (volume = 1400 cm^3^) it would have a cortical surface of only about 480 cm^2^. The actual surface area of the human cortex, however, is about 2000 cm^2^, which is more than four times larger than would be predicted assuming geometric similarity, indicating that mammalian brains change their shape by becoming folded as they increase in size.

### Mechanisms of cortical folding

Previous hypotheses about cortical folding have emphasized mechanisms intrinsic to the cortical gray matter (for reviews, see Hofman, 1989; Bayly et al., 2014). Van Essen (1997) suggested that extrinsic factors are more important and that tension along axons in the white matter is the primary driving force for cortical folding. By keeping the aggregate length of axonal and dendritic wiring low, tension should contribute to the compactness of neural circuitry throughout the cortex. Despite the many attempts to clarify the mechanical basis of cortical folding the process remains incompletely understood.

Recently, Herculano-Houzel and colleagues have found that connectivity and cortical folding are directly related across species and that a simple model based on a white matter-based mechanism may account for increased cortical folding in the primate cerebral cortex (Herculano-Houzel et al., 2010; Mota and Herculano-Houzel, 2012; Ribeiro et al., 2013). They argue that the mechanical tension generated by the pattern of connectivity of fiber bundles traveling through white matter may account for the observed pattern of cortical surface convolutions. The authors propose the degree of tension, taken as directly proportional to the morphological characteristics of the fiber bundle (i.e., axonal length and average cross-sectional area, and the proportion of efferent neurons), determines how much the cortical surface folds inwards.

This model is used to explain how surface convolutions vary with brain size and how gray matter thickness varies. Thus, the local wiring and cortical folding is a simple strategy that helps to fit the large sheetlike cortex into a compact space and keeps cortical connections short. An important evolutionary advantage of this design principle is that it enables brains to be more compact and faster with increasing size (Harrison et al., 2002; Karbowski, 2003).

### Scaling of the primate neocortex

During the past decades considerable progress has been made in explaining the evolution of the cerebral cortex in terms of physical and adaptive principles (see e.g., Macphail and Bolhuis, 2001; Lefebvre, 2012; Roth and Dicke, 2012). In addition, a quantitative approach to the comparative morphology of the brain has made it possible to identify and formalize empirical regularities in the diversity of brain design, especially in the geometry of the cortex (e.g., Hofman, 1989, 2012; Changizi, 2001, 2007; Clark et al., 2001).

**Figure 3:**
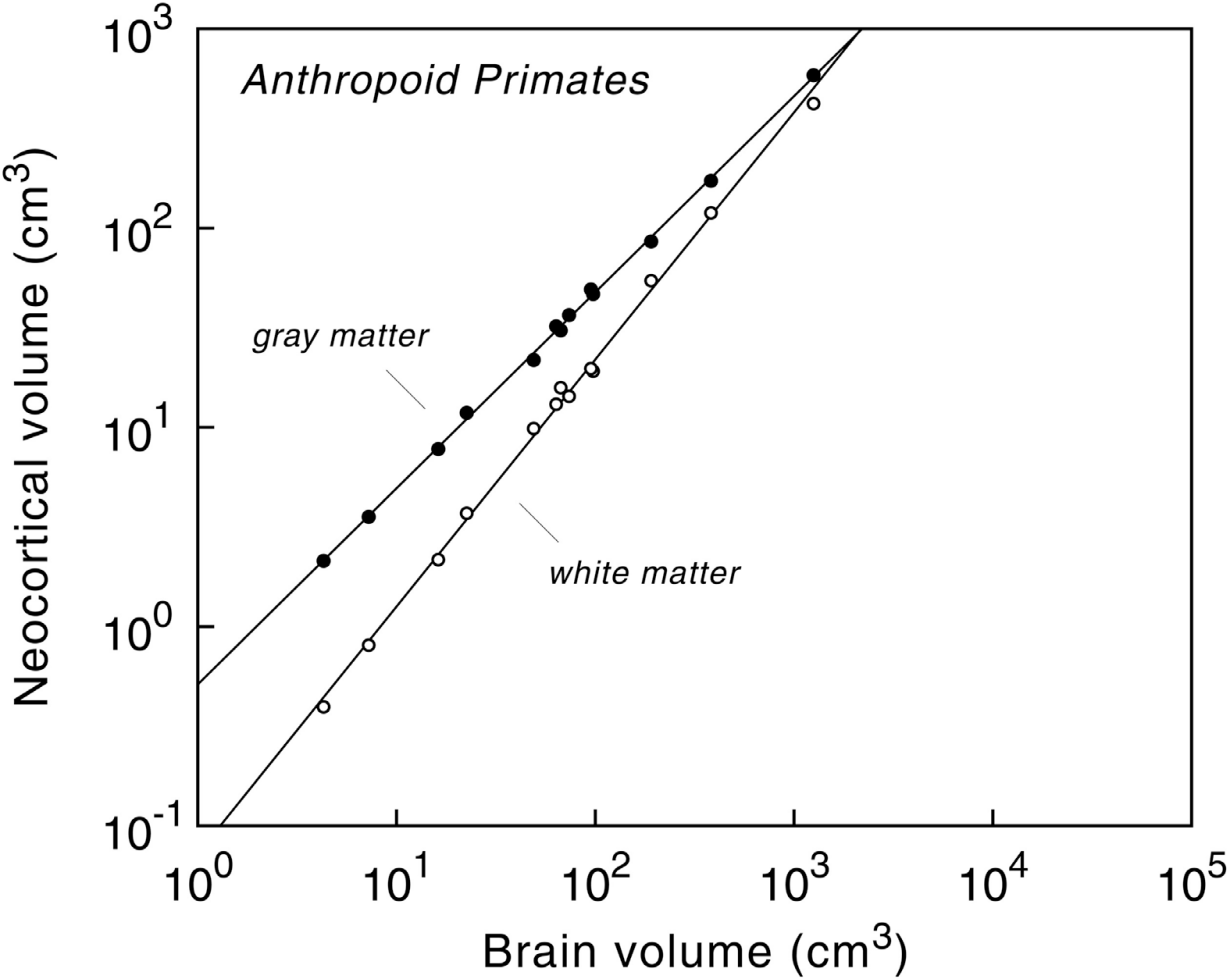
**Volumes of cerebral gray and white matter as a function of brain volume in anthropoid primates, including humans.** Logarithmic scale. The slopes of the regression lines are 0.985 ± 0.009 (gray matter) and 1.241 ± 0.020 (white matter). Note the difference in the rate of change between gray matter (“neural elements”) and white matter (“neural connections”) as brain size increases. Reproduced with permission from Hofman (2001b).

Analysis of the cerebral cortex in anthropoid primates, for example, revealed that the volume of the neocortex is highly predictable from absolute brain size (Hofman, 1989; Finlay and Darlington, 1995; Zhang and Sejnowski, 2000; Finlay et al., 2001; Hofman and Falk, 2012). The volume of the cortical gray matter, containing local networks of neurons that are wired by dendrites and mostly non-myelinated axons, is basically a linear function of brain volume, whereas the mass of long-range axons, forming the underlying white matter volume, increases disproportionately with brain size (Figure 3). As a result, the volume of gray matter expressed as a percentage of total brain volume is about the same for all anthropoid primates.

**Figure 4:**
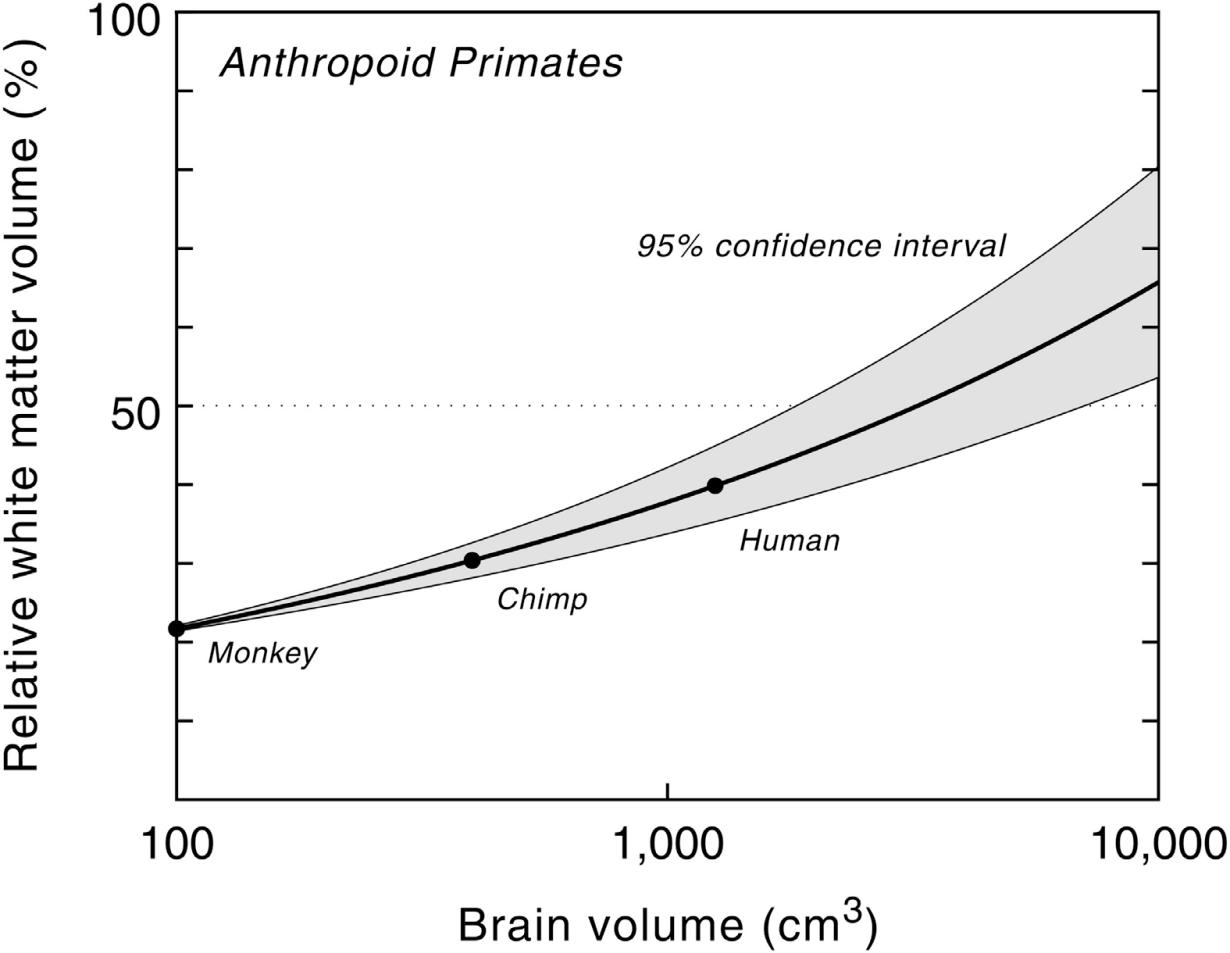
**Relative white matter volume as a function of brain volume in anthropoid primates.** Semilogarithmic scale. The proportion of white matter increases with brain size, from 22% in a monkey brain of 100 cm^3^ to about 65% in a hypothetical primate with a brain size of 10,000 cm^3^. Modified with permission from Hofman (2008).

The relative white matter volume, on the other hand, increases with brain size, from 9% in pygmy marmosets (*Cebuella pygmaea*) to about 35% in humans, the highest value in primates (Hofman, 1989). The non-linear nature of this process is further emphasized by plotting the relative volume of white matter as a function of brain size (Figure 4). The high correlation between both variables ensures that the curve, and its confidence limits, can be used for predictive purposes to estimate the volume of white matter relative to brain volume for a hypothetical primate. The model, for example, predicts a white matter volume of about 1470 cm^3^ for an anthropoid primate with a brain volume of 3000 cm^3^ (Hofman, 2001b, 2012). In other words, in such a large brained primate, white matter would comprise about half of the entire brain volume, compared to one-third in modern man.

Volumetric measurements of gray and white matter in the neocortex of anthropoid primates have shown that the “universal scaling law” of neocortical gray to white matter applies separately for frontal and non-frontal lobes and that changes in the frontal (but not non-frontal) white matter volume are associated with changes in other parts of the brain, including the basal ganglia, a group of subcortical nuclei functionally linked to executive control (Smaers et al., 2010; Sherwood et al., 2012; Sherwood and Smaers, 2013). These comparative analyses indicate that the evolutionary process of neocorticalization in primates is mainly due to the progressive expansion of the axonal mass that implement global communication, rather than to the increase in the number of cortical neurons and the importance of high neural connectivity in the evolution of brain size in anthropoid primates.

## Organization of the cerebral cortex

Evolutionary changes in the cerebral cortex have occurred mainly parallel to the cortical surface (tangentially) and have been sharply constrained in the vertical (radial) dimension, which makes it especially well suited for the elaboration of multiple projections and mapping systems. A mosaic of functionally specialized areas have indeed been found in the mammalian cortex, some of the functions being remarkably diverse (Kaas, 2000, 2008, 2012; Schoenemann, 2006; Krubitzer, 2007; Krubitzer and Dooley, 2013; Ribeiro et al., 2013). At the lower processing levels of the cortex, these maps bear a fairly simple topographical relationship to the world, but in higher areas precise topography is sacrificed for the mapping of more abstract functions. Here, selected aspects of the sensory input are combined in ways that are likely to be relevant to the animal. Human brains, in particular, are distinguished not only by their size but also by a greater proportion of their cortical surface allocated to higher-order association cortex rather than primary sensory and motor areas (Van Essen and Dierker, 2007; Glasser et al., 2013). This observation suggests that relatively more of the human cerebral cortex is dedicated to conceptual as opposed to perceptual and motor processing.

Using modern anatomical tracing methods, physiological recordings and mapping studies it has been established that each sensory modality is mapped several times in different areas, with about a dozen representations of the visual world and a half a dozen each of auditory inputs and somatosensory sensations. In fact, the maps differ in the attributes of the stimulus represented, in how the field is emphasized, and in the types of computations performed. Clearly, the specifications of all these representations means that functional maps can no longer be considered simply as hard-wired neural networks. They are much more flexible than previously thought and are continually modified by feedback and lateral interactions. These dynamic changes in maps, which seem likely to result from local interactions and modulations in the cortical circuits, provide the plasticity necessary for adaptive behavior and learning. Although species vary in the number of cortical areas they posses, and in the patterns of connections within and between areas, the structural organization of the primate neocortex is remarkably similar.

### Cortical network circuitry

The tremendous increase in the cortical surface without a comparable increase in its thickness during mammalian evolution has been explained in the context of the radial-unit hypothesis of cortical development (for reviews, see Rakic, 2007, 2009). According to this model, neocortical expansion is the result of changes in proliferation kinetics that increase the number of radial columnar units without changing the number of neurons within each unit significantly. Therefore the evolutionary expansion of the neocortex in primates is mainly the result of an increase in the number of radial columns.

The widespread occurrence of these neocortical columns, furthermore, qualifies them to be considered as fundamental building blocks in neural evolution (Mountcastle, 1997; Rockland, 2010; Buxhoeveden, 2012). It has become evident that these cortical circuits integrate at higher levels of information processing, as a result of the hierarchical organization of the brain, thus enabling the system to combine dissimilar views of the world. It implies that if we seek the neural basis of biological intelligence, including mind-like properties and consciousness, we can hardly localize it in a specific region of the brain, but must suppose it to involve all those regions through whose activity an organism is able to construct an adequate model of its external world, perhaps it may even encompass the entire neo- and subcortical network. Although the details of the interpretation of the columnar organization of the neocortex are still controversial (for recent reviews, see Da Costa and Martin, 2010; Rockland, 2010), it is evident that the potential for brain evolution results not from the unorganized aggregation of neurons but from cooperative association by the self-similar compartmentalization and hierarchical organization of neural circuits and the invention of fractal folding, which reduces the interconnective axonal distances.

Comparative studies furthermore indicate that variability in subtle subcomponents of the columnar organization in human and non-human primates, such as the composition of the interneuron subtypes, are a primary source of interspecific differences in minicolumn morphology among species (Raghanti et al., 2010; Sherwood et al., 2012). Humans deviate from other primates in having a greater width of minicolumns in specific cortical areas, especially in the prefrontal cortex, owing to constituents of the peripheral neuropil space (Buxhoeveden and Casanova, 2002; Semendeferi et al., 2011). These findings support the idea that human evolution, after the split from the common ancestor with chimpanzees, was accompanied by discrete modifications in local circuitry and interconnectivity of selected parts of the brain (see e.g., Semendeferi et al., 2002; Allen, 2009; Teffer and Semendeferi, 2012). The differences in columnar diameter among primates, however, is only minor compared to the dramatic variation in overall cortex size. Thus it seems that the main cortical change during evolution has presumably been an increase in the number, rather than the size of these neural circuits.

### Cortical circuits: architecture and wiring

The evolutionary expansion of the neocortex in primates, as we have seen, is mainly the result of an increase in the number of radial columns, of which the architecture may have been under selective evolutionary pressure in different mammalian lineages in response to encephalization and specializations of cognitive abilities. We are beginning to understand some of the geometric and biophysical constraints that have governed the evolution of these neural networks (see e.g., Chklovskii et al., 2002; Klyachko and Stevens, 2003; Laughlin and Sejnowski, 2003; Rockland, 2010). To operate efficiently within these constraints, nature has optimized the structure and function of these processing units with design principles similar to those used in electronic devices and communication networks. In fact, the basic structural uniformity of the cerebral cortex suggests that there are general architectural principles governing its growth and evolutionary development (Cherniak, 1995, 2012; Rakic, 1995; Hofman, 2001a, 2007).

Recent studies in primates have shown that the number of neurons underneath a unit area of cortical surface is not constant and varies linearly with neuronal density, a parameter that is neither related to cortical size nor to the total number of neurons (Herculano-Houzel et al., 2008; Wang et al., 2008; Herculano-Houzel, 2009). These studies indicate that the cortical column varies both in size and number of neurons, which is in accordance with predictions based on computational models (Hofman, 1985b). Indeed, comparative morphological differences between cortical areas and species cast doubt on the notion of a universal cortical module or minicolumn (DeFelipe et al., 2002). Furthermore, a review of extrinsic thalamocortical and intrinsic excitatory pathways in the rodent barrel cortex by Feldmeyer (2012), shows that neurons and their interconnections are not static but are dynamically regulated by behavioral state and synaptic plasticity (see also Budd and Kisvárday, 2012).

## Principles of neural wiring

Studies in mammals have shown that in species with convoluted brains the mass of interconnective nerve fibers, forming the underlying white matter, is proportional to the 1.28 power of brain volume (Hofman, 1988, 1991), meaning that the cortical white matter is a fractal system. As a result, the total cortical surface area, including all gyri and sulci, scales approximately as the 2/3 power of the white matter volume.

In other words, the surface area of the cerebral cortex, and with that the total number of cortical columns, is geometrically similar with the amount of white matter, i.e., with the number and length of the interconnective nerve fibers. In small species with non-convoluted brains a similar relationship was found between the cortical surface area and the mass of myelinated nerve fibers (Hofman, 1991, see also Hofman, 2012). A fractal dimension of *D* = 2.70, as found for convoluted brains (Mandelbrot, 1982; Hofman, 1991), suggests a high degree of parallel processing to take place in the cerebral cortex and emphasizes the processing and/or transfer of information across cortical regions in highly corticalized mammals, such as monkeys and apes, rather than within regions. To reach the state of integral parallelism in which each neural component has its own terminal, the length and number of the interconnective axons must be reduced in order to set limits to the axonal mass.

Therefore the most obvious problem imposed by large brains is increasing distances among the neuronal somata of functionally related regions and the inevitable lengthening of their essential communication lines, the axons. Importantly, the axonal length and volume increase much more rapidly than the number of neurons. Furthermore, a proportional increase of neurons and connections would inevitably lead to a rapid increase of synaptic path length, defined as the average number of monosynaptic connections in the shortest path between two neurons (Watts and Strogatz, 1998; Buzsáki and Draguhn, 2004; Sporns et al., 2004). So that the path length can be maintained, short cut connections can be inserted, resulting in small-world- and scale-free-type networks (Sporns et al., 2007; Bullmore and Sporns, 2012).

Although such a solution can effectively decrease path length within the neocortex, the increased lengths of the axons and the associated increased travel time of the action potentials still pose serious problems. As compensation for these excessive delays, axon caliber and myelination should be increased (Innocenti et al., 2013). An indication that larger brains deploy both more shortcuts (long-range connections) and larger-caliber axons is that the volume of the white matter increased at 4/3 power of the volume of gray matter during the course of evolution. Although the white matter occupies only 6% of the neocortical volume in hedgehogs, it exceeds 40% in humans (Hofman, 1988; Herculano-Houzel, 2009).

Wen and Chklovskii (2005) have shown that the competing requirements for high connectivity and short conduction delay may lead naturally to the observed architecture of the mammalian neocortex. Obviously, the brain functionally benefits from high synaptic connectivity and short conduction delays. A magnetic resonance imaging study, furthermore, focusing specifically on the prefrontal cortex, has shown that the volume of the white matter underlying prefrontal areas is disproportionately larger in humans than in other primates (Schoenemann et al., 2005). It suggests that the connectional elaboration of the prefrontal cortex, which mediates such important behavioral domains as planning, aspects of language, attention and social and temporal information processing, has played a key role in human brain evolution.

Although the frontal lobe as a whole has not been differentially enlarged throughout human evolution (Semendeferi and Damasio, 2000; Teffer and Semendeferi, 2012), there is increasing evidence for its reorganization, as some regions with known functional correlates are either bigger or smaller in the human brain than expected when compared with the same region in great apes. Comparative studies, for example, suggest that the human prefrontal cortex differs from that of closely related primate species less in relative size than it does in organization (for a review, see Teffer and Semendeferi, 2012). Specific reorganizational events in neural circuitry may have taken place either as a consequence of adjusting to increases in size or as adaptive responses to specific selection pressures. It appears that the evolution of the human brain was accompanied by discrete modifications in local circuitry and interconnectivity of selected parts of the brain and that these species-specific adaptations may effect these parts differently (Striedter, 2005; Schoenemann, 2006). But the similarity in brain design among primates, including humans, indicates that brain systems among related species are internally constrained and that the primate brain could only evolve within the context of a limited number of potential forms.

### Neocortical wiring

In the neocortex, billions of neurons are interconnected via a massive yet highly organized network of axonal and dendritic wiring. This wiring enables both near and distant neurons to coordinate their responses to external stimulation. Specific patterns of cortical activity generated within this network have been found to correlate with cognitive and perceptual functions (Wang, 2010). Understanding the organizing principles of cortical wiring, therefore, represents a central goal toward explaining human cognition and perception in health and disease. Despite more than a century of endeavor, however, the organizing principles and function of cortical connectivity are not well understood (see e.g., Douglas and Martin, 2004; Bohland et al., 2009; Budd and Kisvárday, 2013)

Recent network studies, using diffusion tensor imaging (DTI), have demonstrated that not only the neurons in the neocortex are structurally and functionally highly organized, but that it also holds for the wiring of the brain (Van den Heuvel and Sporns, 2011; Wedeen et al., 2012). The interconnecting white matter axonal pathways are not a mass of tangled wires, as thought for a long time, but they form a rectilinear three-dimensional grid continuous with the three principal axes of development. The topology of the brain's long-range communication network looks like a 3-D chess board with a number of highly connected neocortical and subcortical hub regions. Structural connectivity networks, as defined by DTI, have identified a common hub in the medial parietal cortex of humans, chimpanzees, and macaque monkeys. However, the apparent lack of medial prefrontal hubs in humans that are present in chimpanzees and macaque monkeys, coupled with evidence of increased gyrification in human prefrontal cortex, suggests important evolutionary changes in the connectivity of human prefrontal cortex (Preuss, 2011; Li et al., 2013; for a review, see Rilling, 2014).

The competing requirements for high connectivity and short conduction delay may lead naturally to the observed architecture of the human neocortex. Obviously, the brain functionally benefits from high synaptic connectivity and short conduction delays. The design of the primate brain is such that it may perform a great number of complex functions with a minimum expenditure of energy and material both in the performance of the functions and in the construction of the system. In general there will be a number of adequate designs for an object, which, for practical purposes, will all be equivalent.

We have shown that in species with convoluted brains the fraction of mass devoted to wiring seems to increase much slower than that needed to maintain a high degree of connectivity between the neural networks (Hofman, 2007, 2012). These findings are in line with a model of neuronal connectivity (Deacon, 1990; Ringo, 1991) which says that as brain size increases there must be a corresponding fall in the fraction of neurons with which any neuron communicates directly. The reason for this is that if a fixed percentage of interconnections is to be maintained in the face of increased neuron number, then a large fraction of any brain size increase would be spent maintaining such degree of wiring, while the increasing axon length would reduce neural computational speed (Ringo et al., 1994). The human brain, for example, has an estimated interconnectivity of the order of 10^3^, based on data about the number of modular units and myelinated nerve fibers (Hofman, 2012). This implies that each cortical module is connected to a thousand other modules, and that the mean number of processing steps, or synapses, in the path interconnecting these modules, is about two.

Herculano-Houzel et al. (2010) have shown that in primates the mass of the white matter scales linearly across species with its number of non-neuronal cells, which is expected to be proportional to the total length of myelinated axons in the white matter. Decreased connectivity in the brain is compatible with previous suggestions that neurons in the cerebral cortex are connected as a small-world network and should slow down the increase in global conduction delay in cortices with larger numbers of neurons (Sporns et al., 2004, 2007; Wang et al., 2008; Bohland et al., 2009).

Recently, Perin et al. (2013) examined theoretically the role of arbor morphology and neuronal density on the emergence of spatially overlapping clusters of recurrently connected cortical neurons. These clusters are generated by repeatedly applying the common neighbor wiring rule until the network structure stabilizes. In this rule the probability of connection between a pair of neurons is proportional to the number of connections they have in common (Perin et al., 2011). The authors report arbor extent limits the size and number of neuronal clusters, which they propose could form innate, elemental cortical groupings.

**Figure 5:**
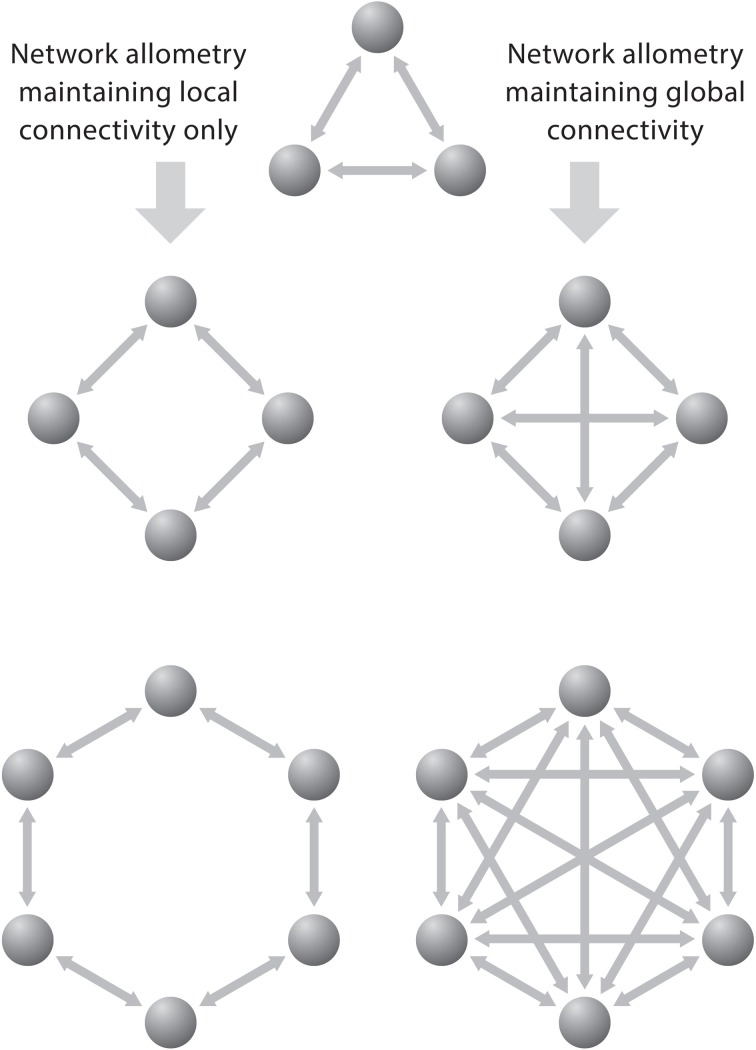
**The problem of network allometry is represented by two neural circuits that exhibit local and global connectivity, respectively.** These diagrams depict that the number of bilateral connections (C) grows much faster than the number of units (U) in a fully connected network: *C* = U (U−1) than in a binary system, where the growth of connections is a linear function of the number of units. Reproduced with permission from Hofman (2008).

To group neurons into clusters interacting over relatively short distances allows these groups to inform as many adjacent clusters of neurons about the state of the “emitting” cluster with as little as possible redundancy of information. Figure 5 shows a simple schematic diagram illustrating the effect of increasing the number of functional cortical units on the number of interconnections. When the units are connected to all others by separate fibers and when each additional unit becomes connected with each of the already existing ones, then the number of connections (C) is related to the number of units (U) according to the equation: *C* = U (U−1), which is nearly equivalent to *C* = U^2^. In such a system the number of connections increases much faster than the number of units. Generally, the growth of connections to units is a factorial function of the number of units in a fully connected network and a linear function of the number of units in a minimally connected network.

Once the brain has grown to a point where the bulk of its mass is in the form of connections, then further increases (as long as the same ratio in interconnectivity is maintained) will be unproductive. Increases in number of units will be balanced by decreased performance of those units due to the increased conduction time. This implies that large brains may tend to show more specialization in order to maintain processing capacity. Indeed, an increase in the number of distinct cortical areas with increasing brain size has been reported (Kaas, 2000, 2012; Striedter, 2005). It may even explain why large-brained species may develop some degree of brain lateralization as a direct consequence of size. If there is evolutionary pressure on certain functions that require a high degree of local processing and sequential control, such as linguistic communication in human brains, these will have a strong tendency to develop in one hemisphere.

## Biological limits to information processing

Although the cerebral cortex is not the only brain structure which was selected for in evolution to expand, as a result of growing environmental pressure for more sophisticated cognitive abilities, it has played a key role in the evolution of information processing in the mammalian brain. The primate cortex, as we have seen, has evolved from a set of underlying structures that constrain its size, and the amount of information it can store and process. If the ability of an organism to process information about its environment is a driving force behind evolution, then the more information a system, such as the brain, receives, and the faster it can process this information, the more adequately it will be able to respond to environmental challenges and the better will be its chances of survival (Hofman, 2003). The limit to any intelligent system therefore lies in its abilities to process and integrate large amounts of sensory information and to compare these signals with as many memory states as possible, and all that in a minimum of time. It implies that the functional capacity of a neuronal structure is inherently limited by its neural architecture and signal processing time (see e.g., Hofman, 2001a; Laughlin and Sejnowski, 2003; Changizi and Shimojo, 2005).

The processing or transfer of information across cortical regions, rather than within regions, in large-brained primates can only be achieved by reducing the length and number of the interconnective axons in order to set limits to the axonal mass. The *number* of interconnective fibers can be reduced, as we have seen, by compartmentalization of neurons into modular circuits in which each module, containing a large number of neurons, is connected to its neural environment by a small number of axons. The *length* of the interconnective fibers can be reduced by folding the cortical surface and thus shortening the radial and tangential distances between brain regions. Local wiring—preferential connectivity between nearby areas of the cortex—is a simple strategy that helps keep cortical connections short. In principle, efficient cortical folding could further reduce connection length, in turn reducing white matter volume and conduction times (Young, 1993; Scannell et al., 1995; Chklovskii et al., 2004; Buzsáki et al., 2013). Thus the development of the cortex does seem to coordinate folding with connectivity in a way that could produce smaller and faster brains.

Wang et al. (2008) have shown that there are functional trade-offs in white matter axonal scaling in mammals. They found that the composition of white matter shifts from compact, slow-conducting, and energetically expensive unmyelinated axons to large, fast-conducting, and energetically inexpensive myelinated axons. The fastest axons have conduction times of 1–5 ms across the neocortex and <1 ms from the eye to the brain, suggesting that in select sets of communicating fibers, large brains reduce transmission delays and metabolic firing costs at the expense of increased volume. Delays and potential imprecision in cross-brain conduction times are especially great in unmyelinated axons, which may transmit information via firing rate rather than precise spike timing. In the neocortex, axon size distributions can account for the scaling of per-volume metabolic rate and suggest a maximum supportable firing rate, averaged across all axons, of 7 ± 2 Hz. Clearly, the white matter architecture must follow a limited energy budget to optimize both volume and conduction time (for a review, see Buzsáki et al., 2013).

Another way to keep the aggregate length of axonal and dendritic wiring low, and with that the conduction time and metabollic costs, is to increase the degree of cortical folding. A major disadvantage of this evolutionary strategy, however, is that an increase in the relative number of gyri can only be achieved by reducing the gyral width. At the limit, the neurons in the gyri would be isolated from the remainder of the nervous system, since there would no longer be any opening for direct contact with the underlying white matter. Prothero and Sundsten (1984) therefore introduced the concept of the gyral “window,” which represents the hypothetical plane between a gyrus and the underlying white matter through which nerve fibers running to and from the gyral folds must pass. According to this hypothesis, there would be a brain size where the gyral “window” area has an absolute maximum. A further increase in the size of the brain beyond that point, i.e., at 2800 cm^3^, would increase the cortical surface area, but the “window” would decrease, leading to a lower degree of neuronal integration and an increase in response time.

**Figure 6:**
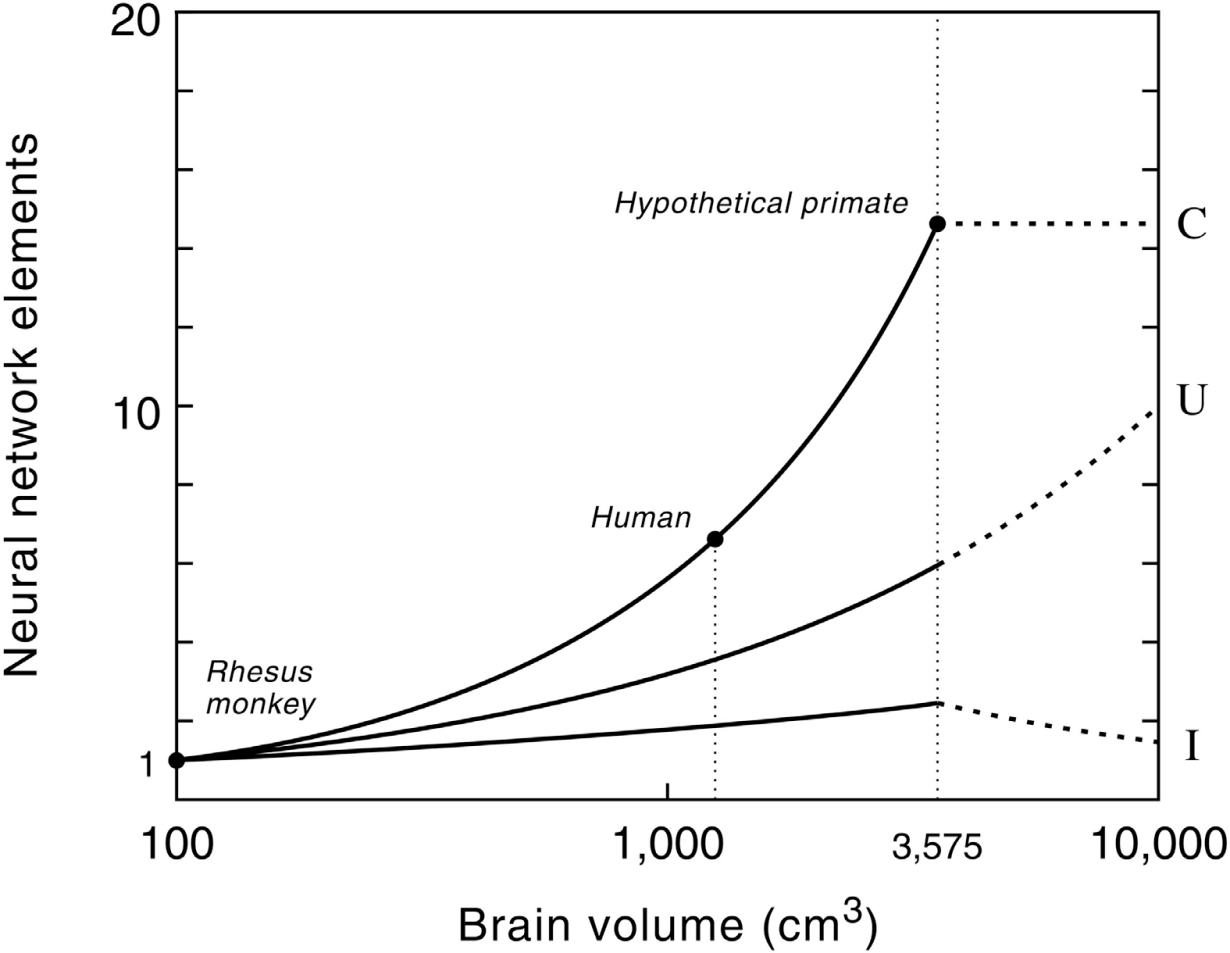
**The number of connections (C), cortical processing units (U) and level of interconnectivity (I) in the primate neocortex as a function of brain size.** Semi-logarithmic scale. Values are normalized to one at a brain volume of 100 cm^3^, the size of a monkey brain. Note that the number of myelinated axons increases much faster than the number of cortical processing units (see also Figure 5). The human cerebrum, for example, contains 6 times more myelinated axons than that of a rhesus monkey, whereas the number of cortical processing units is only 3 times larger. Dashed lines show the potential evolutionary pathway of these neural network elements in primates with very large brains, i.e., beyond the hypothetical upper limit of the brain's processing power (see text and Figure 7). Note that a further exponential growth in the number of cortical processing units, without an increase in the number of connections, will lead to a decrease in connectivity between these units and thus to more local wiring. Reproduced with permission from Hofman (2012).

The remarkably high correlation between gray matter, white matter and brain size in anthropoid primates ensures that the proposed model can be used for predictive purposes to estimate the volume of white matter relative to brain volume for a hypothetical primate (Hofman, 2001b). Model studies of the growth of the neocortex at different brain sizes, using a conservative scenario, revealed that with a brain size of about 3500 cm^3^ the total volume of the subcortical areas (i.e., cerebellum, brain stem, diencephalon, etc.) reaches a maximum value (Figure 6). Increasing the size of the brain beyond that point, following the same design principle, would lead to a further increase in the size of the neocortex, but to a reduction of the subcortical volume. Consequently, primates with very large brains (e.g., over 5 kg) may have a declining capability for neuronal integration despite their larger number of cortical neurons.

## Limits to human brain evolution

A progressive enlargement of the hominid brain started about 2.5 million years ago, probably from a bipedal, australopithecine form with a brain size comparable to that of a modern chimpanzee (see e.g., Falk, 2007, 2012; Robson and Wood, 2008; De Sousa and Cunha, 2012; Schoenemann, 2013). Since then, a threefold increase in endocranial volume has taken place, leading to one of the most complex and efficient structures in the animated universe, the human brain. Explanations for the evolution of the human brain are mainly focusing on selection pressures of the physical environment (climate, diet, food availabilty) and those of the social environment (group size, coalition formation, parental care). Although attempts have been made to discriminate between ecological and social theories (see e.g., Leigh, 2004; Dunbar and Shultz, 2007a,b; Lefebvre, 2012; Roth and Dicke, 2012; Willemet, 2013) there has been little effort to develop an explanatory framework that integrates the many social, ecological, and life history correlates of brain size that have been identified. Obviously, they all play a role in explaining the marked differences in brain size between humans and apes, but in which way and to what extent is far from clear at this moment. We will need better studies of cognition and behavior, along with comparative brain studies, to answer these questions.

Despite these difficulties in explaining the selection pressures of the evolution of the human brain, comparative neuroimaging studies in primates have identified the underlying neural substrate and unique features of the human brain (for a review see, Rilling, 2014). These studies, for example, have clarified how the dramatic differences in brain size between humans and chimpanzees develop. First, human brains are already twice as large as chimpanzee brains from an early point in gestation (16 weeks). Although both show an increase in growth velocity at this time, they diverge sharply at 22 weeks of gestation, when human brain growth continues to accelerate, whereas chimpanzee brain growth decelerates. Finally, during early infancy, humans experience a very rapid increase in white matter volume that significantly exceeds that found in chimpanzees (see e.g., Sakai et al., 2013).

In view of the central importance placed on brain evolution in explaining the success of our species, one may wonder whether there are physical limits that constrain its processing power and evolutionary potential. The human brain has evolved from a set of underlying structures that constrain its size, and the amount of information it can store and process. In fact, there are a number of related factors that interact to limit brain size, factors that can be divided into two categories: (1) energetic constraints and (2) neural processing constraints (see e.g., Hofman, 2001b, 2012; Wang et al., 2008; Herculano-Houzel, 2009).

### Energetic limits

The human brain generates about 15 watts (W) in a well insulated cavity of about 1500 cm^3^. From an engineering point of view, removal of sufficient heat to prevent thermal overload could be a significant problem. But the brain is actively cooled by blood and not simply by heat conduction from the surface of the head. So the limiting factor is how fast the heat can be removed from the brain by blood flow. It has been suggested by Falk (1990) and others that the evolution of a “cranial radiator” in hominids helped provide additional cooling to delicate and metabolically expensive parts of the brain, such as the cerebral cortex. This vascular cooling mechanism would have served as a “prime releaser” that permitted brain size to increase dramatically during human evolution. So, to increase cooling efficiency in a larger brain, either the blood must be cooler when it first enters the structure, or the flow-rate must be increased above current levels.

Another factor related to blood flow has to do with the increasing energy requirements of a larger brain, a problem that is exacerbated by the high metabolic cost of this organ. It is unlikely, however, that the rate of blood flow or the increasing volume used by the blood vessels in the brain—in human about 4%—constrain its potential size. A bigger brain is metabolically possible because our cardiovascular system could evolve to transport more blood at greater pressure to meet the increased demand. This should not be taken to imply that thermal and metabolic mechanisms play no role at all in setting limits to brain size. Ultimately, energetic considerations will dictate and restrict the size of any neuron-based system, but as theoretical analyses indicate, thermal and metabolic factors alone are unlikely to constrain the potential size of our brain until it has increased to at least ten times its present size (Cochrane et al., 1995).

The same holds for extrinsic developmental constraints that have to do with pelvic anatomy (related to bipedalism), parturition, and maternal and fetal mortality. Although these factors are relevant in human evolution it is unlikely that they are setting limits to human brain growth. In primates, for example, selection for increased brain size is associated with precociality, resulting in a cascade of evolutionary effects including increased birth space and single births, prolonged periods of postnatal development, a proportionate delay in maturation and reproductive rate and, thus, an increase in “generation time” (Leigh, 2004). It means that natural selection operates on brain size at the expense of growth and reproduction, which could explain its correlation with life span (Hofman, 1993). This evolutionary strategy is most obvious when considering the evolution of our own species, where there has been a presumed twofold increase in life span associated with a more than threefold increase in brain size in a mere 2.5 million years (Hofman, 1984). So any further expansion of the adult human brain beyond its present size may take place without a radical change in its fetal/neonatal developmental schedule.

### Neural-processing limits

**Figure 7:**
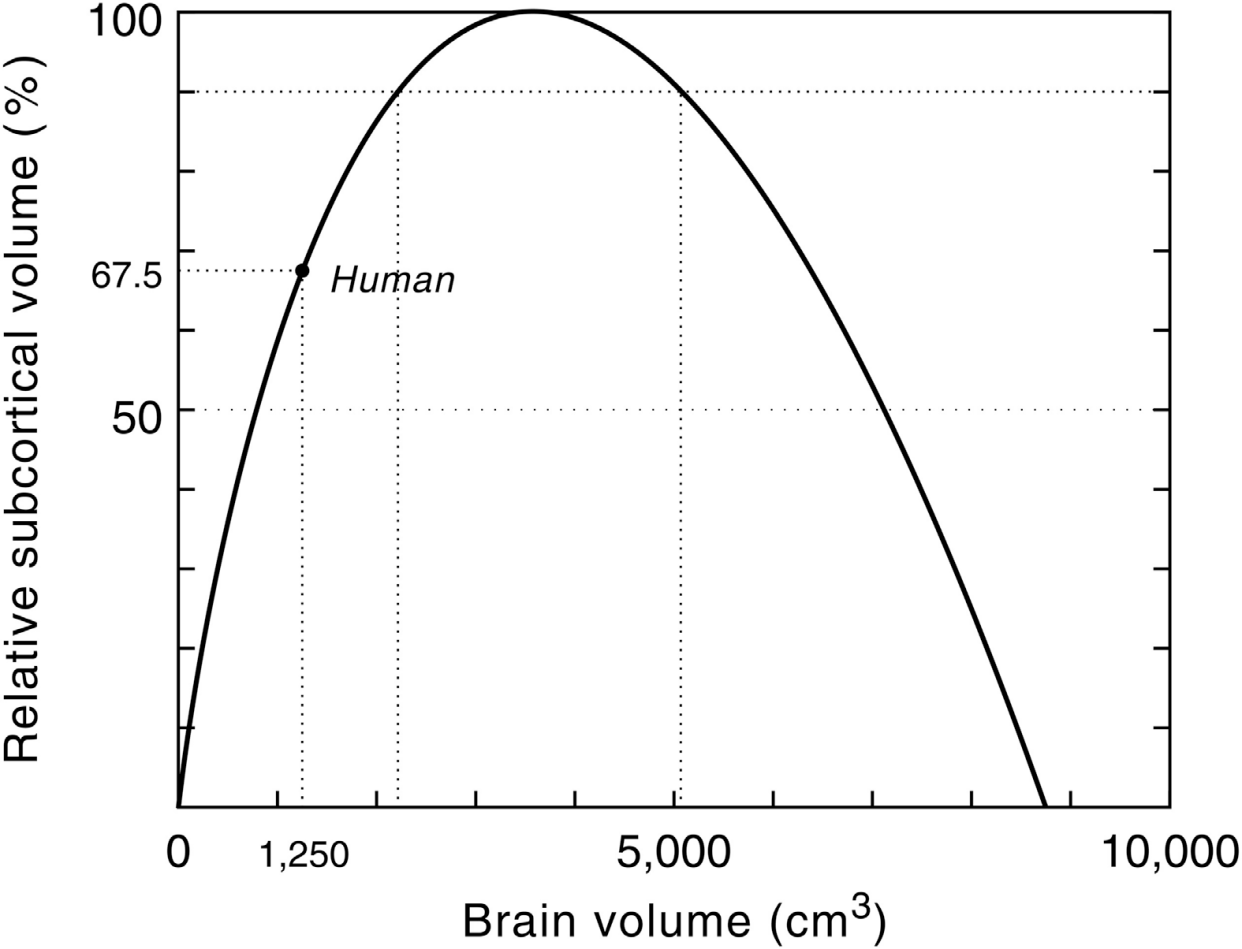
**Relative subcortical volume as a function of brain volume.** The predicted subcortical volume (i.e., brain volume—predicted neocortex volume) must be zero at zero brain size. Likewise, the subcortical volume will be zero when the brain is exclusively composed of cortical gray and white matter. At a brain size of 3575 cm^3^ the subcortical volume has a maximum (see also Figure 6). The maximum simulated value for the subcortical volume (366 cm^3^) is then taken as 100%. The larger the brain grows beyond this critical size, the less efficient it will become. Assuming constant design, it follows that this model predicts an upper limit to the brain's processing power. Modern humans are at about two-third of that maximum. Modified with permission from Hofman (2001b).

The limit to any neural system lies in its ability to process and integrate large amounts of information in a minimum of time and therefore its functional capacity is inherently limited by its neural architecture and signal processing time. The scaling model of the geometry of the neocortex, for example, predicts an absolute upper limit to primate brain size (Hofman, 2001b; Figure 7). Without a radical change in the macroscopic organization of the brain, however, this hypothetical limit will never be approached, since at that point (ca. 8750 cm^3^) the brain would consist entirely of cortical neurons, and their interconnections, leaving no space for any other brain structure.

Cochrane et al. (1995) looked at the different ways in which the brain could evolve to process more information or work more efficiently. They argue that the human brain has (almost) reached the limits of information processing that a neuron-based system allows and that our evolutionary potential is constrained by the delicate balance maintained between conduction speed, duration of the electrical pulse (pulse width), synaptic processing time, and neuron density. By modeling the information processing capability per unit time of a human-type brain as a function of interconnectivity and axonal conduction speed they found that the human brain lies about 20–30% below the optimal, with the optimal processing ability corresponding to a brain about twice the current volume. Any further enhancement of human brain power would require a simultaneous improvement of neural organization, signal processing and thermodynamics. Such a scenario, however, is an unrealistic biological option and must be discarded because of the trade-off that exists between these factors.

Of course, extrapolations based on brain models, such as the ones presented here, implicitly assume a continuation of brain developments that are on a par with growth rates in the past. One cannot exclude the possibility of new structures evolving in the brain, or a higher degree of specialization of existing brain areas, but within the limits of the existing “Bauplan” there does not seem to be an incremental improvement path available to the human brain. At a brain size of about 3500 cm^3^, corresponding to a brain volume two to three times that of modern man, the brain seems to reach its maximum processing capacity. The larger the brain grows beyond this critical size, the less efficient it will become, thus limiting any improvement in cognitive power.

## Concluding remarks

The evolution of the neocortex in primates is mainly characterized by the development and multiplication of clusters of neurons which are strongly interconnected and in physical proximity. Since these clusters of neurons are organized in vertical columns, an increase in the number and complexity of the neuronal networks will be reflected by an expansion of the cortical surface area beyond that expected for geometric similar brains. As a result the cortical surface area fractally evolves into a volume with increasing brain size.

It is evident that the potential for brain evolution results not from the unorganized aggregation of neurons but from cooperative association by the self-similar compartmentalization and hierarchical organization of neural circuits and the invention of cortical folding, which reduces the interconnective axonal distances. The competing requirements for high connectivity and short conduction delay may lead naturally to the observed architecture of the primate neocortex. Obviously, the brain functionally benefits from high synaptic connectivity and short conduction delays. The design of the primate brain is such that it may perform a great number of complex functions with a minimum expenditure of energy and material both in the performance of the functions and in the construction of the system. In general there will be a number of adequate designs for an object, which, for practical purposes, will all be equivalent.

The similarity in brain design among primates, including humans, indicates that brain systems among related species are internally constrained and that the primate brain could only evolve within the context of a limited number of potential forms. It means that internal factors of brain design may be the primary determinants constraining the evolution of the brain and that geometric similarity among species in the functional organization of the brain may be derived from a common ancestor rather than being immediately evolved in response to specific environmental conditions.

### Conflict of interest statement

The author declares that the research was conducted in the absence of any commercial or financial relationships that could be construed as a potential conflict of interest.
